# Case Report: Primary aldosteronism coexisting with adrenal insufficiency in a 73-year-old woman with paradoxical hyponatremia

**DOI:** 10.3389/fimmu.2026.1954159

**Published:** 2026-09-24

**Authors:** Minghong Luo, Zhifang Qin, Ye Tian, Qingxian Tu, Hong Zhao, Xianghui Li, Weiya Zeng, Ran Wang, Min Xu, Qianfeng Jiang

**Affiliations:** 1 Kweichow Moutai Hospital, Moutai Group, Renhuai, Guizhou, China; 2 Department of Cardiovascular Medicine, Guizhou Provincial People’s Hospital, Guiyang, Guizhou, China; 3 The First People’s Hospital of Zunyi (The Third Affiliated Hospital of Zunyi Medical University), Zunyi, Guizhou, China

**Keywords:** Addison’s disease, adrenal insufficiency, autoimmune polyglandular syndrome type 2, autoimmune thyroid disease, case report, primary aldosteronism

## Abstract

**Introduction:**

Primary aldosteronism (PA) and autoimmune polyglandular syndrome type 2 (APS-2) represent opposing ends of adrenal pathology—hyperfunction versus hypofunction. Their coexistence is exceptionally rare and diagnostically challenging.

**Case presentation:**

A 73-year-old woman with a 30-year history of refractory hypertension and recent hyponatremia was diagnosed with PA (aldosterone-to-renin ratio 29.11, positive captopril challenge, left adrenal hyperplasia). Spironolactone controlled blood pressure but paradoxically worsened hyponatremia. Two independent circadian cortisol profiles confirmed persistently low cortisol with preserved normal rhythm (first: 118.65/100.80/50.25 nmol/L; second: 133.34/71.62/39.12 nmol/L at 08:00/16:00/00:00). In contrast, plasma ACTH levels sent to an external laboratory showed no circadian variation (08:00 7.19 pmol/L, 16:00 1.21 pmol/L, 00:00 1.68 pmol/L; reference 1.60-13.90 pmol/L). This discordance-preserved cortisol rhythmicity with absent ACTH rhythm-raised the possibility of a preanalytical artifact rather than secondary adrenal insufficiency, although this inference could not be definitively proven. Physical examination revealed skin hyperpigmentation, and her history of Hashimoto’s thyroiditis supported a clinically probable diagnosis of APS-2 (clinically probable Addison’s disease with autoimmune thyroid disease), given the absence of confirmatory 21-hydroxylase antibody testing and ACTH-stimulation testing.

**Intervention and outcomes:**

Prednisone 5 mg daily was added to her regimen of spironolactone and levothyroxine. Her symptoms resolved, and serum sodium normalized.

**Conclusion:**

This report describes a rare case of primary aldosteronism occurring in the setting of clinically probable APS-2. The discordant cortisol-ACTH pattern serves as a critical reminder that ACTH is highly labile; clinicians must interpret results holistically, integrating clinical context and circadian patterns, and remain mindful of diagnostic uncertainty when confirmatory assays cannot be completed.

## Introduction

Primary aldosteronism (PA) is the most common endocrine cause of secondary hypertension, affecting 5% to 13% of hypertensive patients, and is characterized by autonomous aldosterone secretion, suppressed renin, and typically hypokalemia (1). Autoimmune polyglandular syndrome type 2 (APS-2) is a rare condition defined by the coexistence of Addison’s disease with autoimmune thyroid disease and/or type 1 diabetes mellitus, with an incidence of approximately 1 per 20,000 cases per year (2). The coexistence of PA and APS-2 is exceptionally rare. While one case report described PA with APS-3 (autoimmune thyroid disease without Addison’s) in a 63-year-old woman (3), and another documented concurrent PA and adrenal insufficiency in a 73-year-old man following an abdominal aortic aneurysm rupture (4), to our knowledge, no case of PA with APS-2 has been reported.

We present a unique case where a woman with long-standing hypertension and documented PA was found to have concurrent clinically probable Addison’s disease as part of APS-2, with the diagnostic process nearly derailed by a potential preanalytical ACTH artifact. This case highlights two critical lessons (1): the opposing physiological effects of PA and adrenal insufficiency can mask each other, and (2) ACTH is notoriously labile-preserved cortisol circadian rhythm in the setting of low, non-rhythmic ACTH should raise consideration of sample degradation rather than central pathology, though this remains an unproven inference without laboratory log documentation.

## Case description

A 73-year-old woman presented with a 1-year history of worsening fatigue, palpitations, poor appetite, and poorly controlled hypertension. She had a 30-year history of hypertension, with blood pressure (BP) reaching 180+ mmHg systolic, previously managed with amlodipine 5 mg daily and irbesartan 150 mg daily, though with fluctuating control. Over the preceding year, she had experienced progressive fatigue, palpitations, dyspnea, and anorexia, with documented weight loss of 15 kg. Her medical history included hypothyroidism diagnosed 3 years prior, treated with levothyroxine 50 μg daily, and a CT finding of gastric antral thickening 1 year earlier, which was not investigated further. There was no history of postpartum hemorrhage, malignancy, or long-term corticosteroid use.

On admission, BP was 158/90 mmHg, pulse 48/min, and she had a lean body habitus (weight 44 kg, height 158 cm, BMI 17.6 kg/m²). Physical examination was notable for pallor; no other significant findings were initially observed. Laboratory studies revealed moderate hyponatremia (serum sodium 122 mmol/L) and, on retrospective review, one prior episode of hypokalemia. Electrocardiogram showed sinus bradycardia; chest CT was unremarkable; repeat contrast-enhanced abdominal CT revealed left adrenal thickening (**Figure 1**), suggesting unilateral adrenal hyperplasia.

**Figure 1:**
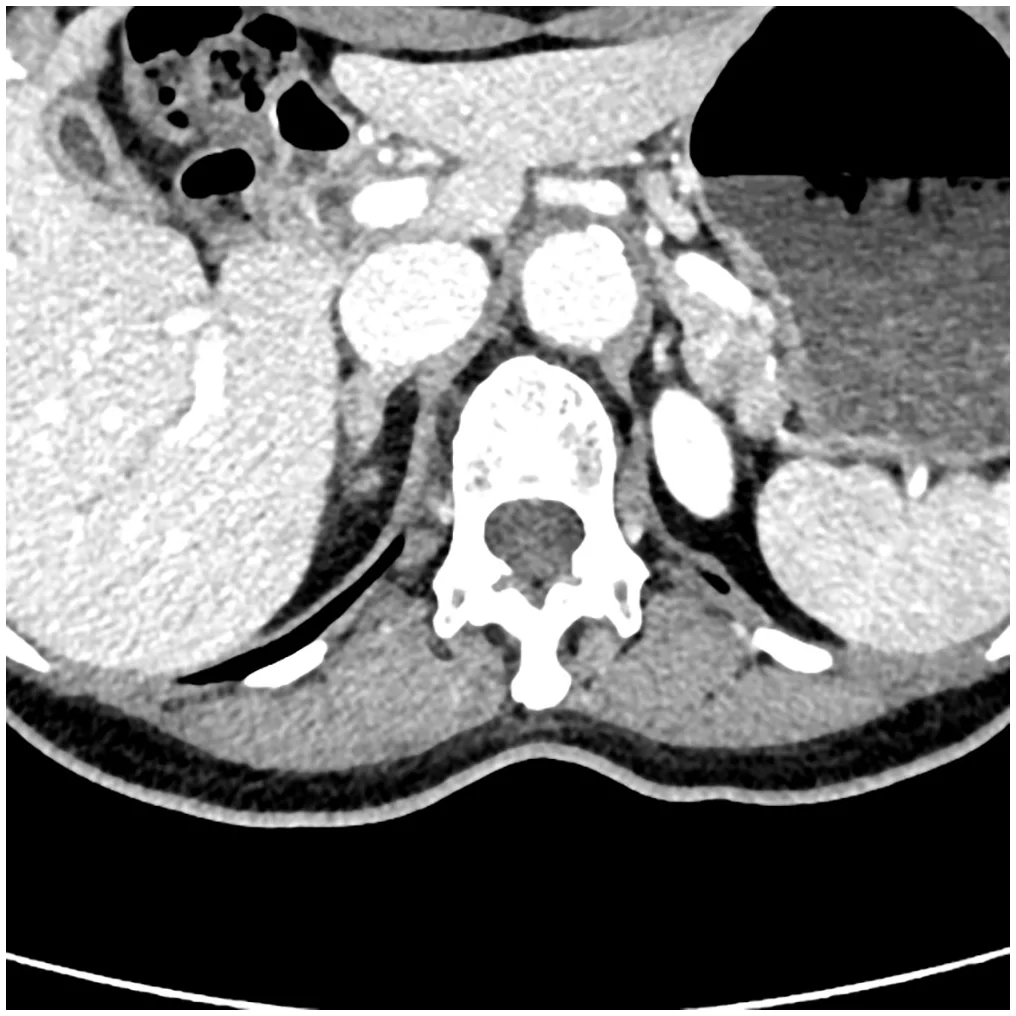
Contrast-enhanced abdominal CT showed left adrenal thickening.

## Diagnostic assessment

Given the refractory hypertension with hyponatremia and prior hypokalemia, secondary hypertension was suspected. The aldosterone-to-renin ratio (ARR) was markedly elevated at 29.11 (aldosterone 15.05 ng/dL, renin 0.52 μIU/mL ↓; reference ARR <3.7) despite the use of amlodipine and irbesartan (which can cause false-negative ARR) and hyponatremia. Captopril challenge test was positive, and left adrenal hyperplasia was confirmed on contrast-enhanced CT. The patient declined adrenal vein sampling. A diagnosis of primary aldosteronism (likely unilateral adrenal hyperplasia) was made (5).

Paradoxically, despite BP improvement and intravenous sodium replacement, the patient’s hyponatremia worsened after spironolactone initiation. A repeated endocrine work-up was performed. Two separate circadian cortisol profiles were obtained. The first profile showed consistently low serum cortisol levels: 08:00 118.65 nmol/L, 16:00 100.80 nmol/L, and 00:00 50.25 nmol/L. The second profile, performed for confirmation, revealed similarly low values: 08:00 133.34 nmol/L, 16:00 71.62 nmol/L, and 00:00 39.12 nmol/L (reference range for morning: 185–624 nmol/L). Notably, both profiles demonstrated a preserved normal circadian rhythm, with peak values in the morning and trough at midnight. This represented a helpful diagnostic clue: preserved rhythmicity made secondary adrenal insufficiency less probable but could not completely exclude it, as cortisol secretion follows a circadian pattern dictated by central suprachiasmatic nucleus inputs to the hypothalamus (6).

However, plasma ACTH levels sent to an external laboratory showed no circadian variation (08:00 7.19 pmol/L, 16:00 1.21 pmol/L, 00:00 1.68 pmol/L; reference 1.60–13.90 pmol/L), which was discordant with the cortisol rhythm. In addition, pituitary MRI was normal, excluding structural lesions. Given the absence of confirmatory 21-hydroxylase antibody testing and ACTH-stimulation testing, the physical examination finding of skin hyperpigmentation (**Figure 2**) and her history of Hashimoto's thyroiditis supported only a clinically probable diagnosis of APS-2 (7, 8).

**Figure 2:**
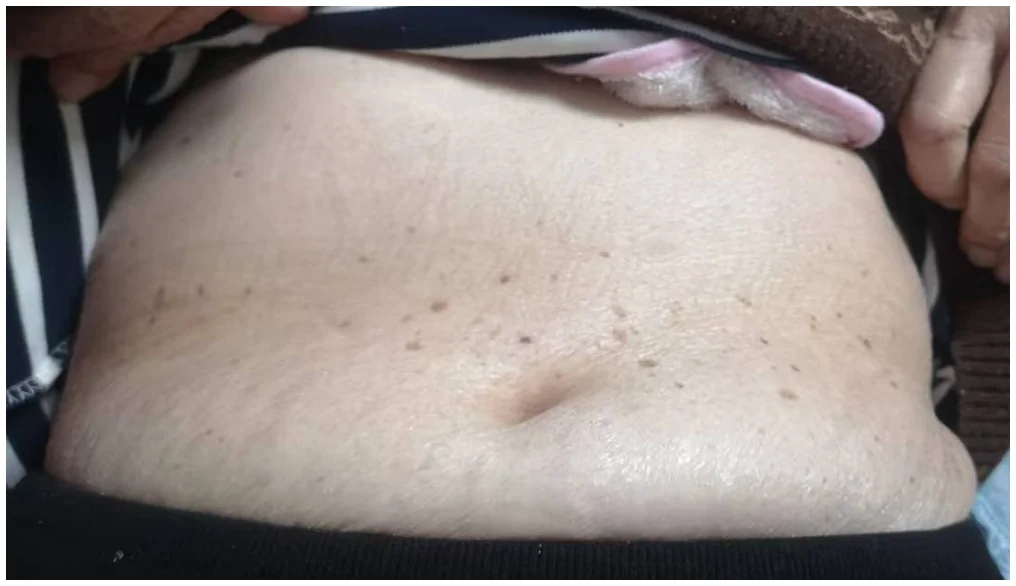
Skin hyperpigmentation in the waistband area, attributable to chronic friction, supporting the diagnosis of primary adrenal insufficiency (Addison’s disease).

Differential diagnoses formally evaluated included: (1) secondary adrenal insufficiency due to hypothalamic-pituitary disease; (2) accelerated cortisol clearance mediated by levothyroxine; (3) other primary adrenal insults such as infectious, infiltrative, or hemorrhagic adrenal injury. Each alternative was judged less probable on aggregate clinical and laboratory grounds, though none could be fully excluded.

## Therapeutic intervention and follow-up

The patient was started on prednisone 5 mg daily, levothyroxine was increased to 75 μg daily, and spironolactone was continued with careful monitoring. At follow-up, the patient’s symptoms improved, and serum sodium normalized.

## Discussion

This case illustrates the diagnostic complexity when two endocrine disorders with opposing physiological effects coexist. Primary aldosteronism typically causes hypertension, hypokalemia, and metabolic alkalosis, whereas Addison’s disease causes hypotension, hyponatremia, hyperkalemia, and metabolic acidosis. In this patient, the aldosterone excess from PA partially counterbalanced the sodium-wasting effects of adrenal insufficiency, resulting in a misleadingly normal or mildly abnormal electrolyte profile.

The diagnostic journey was complicated by several factors. First, the patient’s long-standing hypertension was attributed solely to essential hypertension for decades. Second, the moderate hyponatremia, which should have raised suspicion for adrenal insufficiency, was initially attributed to poor oral intake and the sodium-wasting effect of spironolactone. Third, the use of antihypertensives and the presence of hyponatremia can lower ARR, yet this patient’s ARR remained markedly elevated, masking the underlying Addison’s disease.

A particularly instructive aspect of this case involves the discordant cortisol and ACTH results. The patient’s low cortisol levels were unequivocal-consistent across two separate sampling timepoints (first profile: 118.65/100.80/50.25 nmol/L; second profile: 133.34/71.62/39.12 nmol/L at 08:00/16:00/00:00 respectively)-and confirmed adrenal insufficiency. Yet the HPA axis appeared functionally intact based on two key observations. First, cortisol maintained a normal circadian rhythm, with peak at 08:00 and trough at midnight in both profiles. Cortisol secretion is regulated by the suprachiasmatic nucleus through CRH-ACTH signaling (6). Second, pituitary MRI showed no structural lesions. These observations reduced the probability of secondary (central) adrenal insufficiency but could not definitively rule it out. The incongruent ACTH results-which showed no circadian rhythm (7.19 pmol/L at 08:00, 1.21 pmol/L at 16:00, 1.68 pmol/L at 00:00) and were inappropriately normal-were therefore most consistent with the possibility of preanalytical factors. The absence of the expected physiological surge in ACTH at 08:00, despite two clear cortisol peaks at that time, was particularly telling. If this represented true secondary adrenal insufficiency, cortisol would not have shown a morning peak; if it were primary adrenal insufficiency, ACTH would have been markedly elevated. The pattern was internally inconsistent, pointing to possible sample degradation during transport or storage. We emphasize again that preanalytical degradation remains an inference without verifiable laboratory shipping records.

ACTH is a 39-amino-acid peptide with a half-life of 10–15 minutes and is highly unstable in whole blood due to rapid proteolytic degradation (9, 10). The Endocrine Society guidelines and laboratory protocols emphasize that ACTH samples must be collected in pre-chilled EDTA tubes, placed immediately on ice, centrifuged promptly, and frozen within 1 hour of collection (11, 12). Improper handling can lead to falsely low results. Recent systematic review data confirm that while ACTH remains stable in uncentrifuged EDTA tubes for 6 hours at room temperature and at least 8 hours under refrigerated conditions (9) degradation becomes significant beyond these timeframes. The discordant ACTH results could have misled a clinician into suspecting secondary adrenal insufficiency, prompting unnecessary further investigation or inappropriate therapeutic decisions. This pitfall is well recognized: the clinical interpretation of paired cortisol and ACTH measurements requires careful consideration of preanalytical variables, as ACTH instability is a known source of diagnostic error (9, 10).

The patient had been on a stable dose of levothyroxine (50 μg/day) for three years before admission. Admission tests showed subclinical hypothyroidism (elevated TSH, normal FT3/FT4), indicating slightly insufficient replacement. Although levothyroxine may increase cortisol metabolic clearance and precipitate adrenal crisis in patients with unrecognized adrenal insufficiency (13), this effect is predominantly reported in the context of initiating levothyroxine therapy before glucocorticoid replacement (14). Given stable long-term dosing and euthyroid status, levothyroxine was unlikely to explain sustained low cortisol. CT detected left-sided adrenal thickening represented morphological change related to PA, not Addison’s disease. In autoimmune Addison’s disease, adrenal atrophy is the characteristic CT finding, whereas adrenal glands may appear normal or enlarged in early or non-atrophic stages (15). Adrenal calcification is more frequently observed in tuberculous or long-standing cases (15). Therefore, unilateral thickening represented the PA-related pathology within this dual adrenal pathology clinical scenario (5).

APS-2 is defined by the presence of Addison’s disease with autoimmune thyroid disease and/or type 1 diabetes mellitus (16). The diagnosis requires a high index of suspicion, as many patients present with non-specific symptoms such as fatigue, weight loss, and hyperpigmentation. In our patient, the presence of Hashimoto’s thyroiditis, clinically probable Addison’s disease (supported by low cortisol, hyperpigmentation, low DHEA-S (17–19), and clinical response to steroids), and the exclusion of other causes supported a diagnosis of clinically probable APS-2. The prevalence of APS-2 is approximately 1 in 20,000, with a female predominance (20).

The coexistence of PA and APS-2 is exceptionally rare, and the pathogenic link between these two conditions warrants discussion. Emerging evidence suggests that aldosterone exerts proinflammatory effects that may contribute to the development or exacerbation of autoimmune disorders (21). Aldosterone promotes an inflammatory state characterized by vascular infiltration of immune cells and proinflammatory cytokine production (22). Herrada et al. demonstrated that aldosterone can directly promote CD4^+^ T-cell polarization toward Th17, resulting in the development of organ-specific autoimmune diseases (23).

Krysiak and Okopień first reported the coexistence of PA and Hashimoto’s thyroiditis (APS-3 variant) and demonstrated that surgical removal of an aldosterone-producing adenoma significantly improved thyroid function and reduced thyroid autoantibody titers (24). This provided direct evidence that aldosterone excess exerts a proinflammatory effect via mineralocorticoid receptor activation on immune cells, potentially exacerbating pre-existing autoimmune processes. A recent case report described a 63-year-old woman with PA and APS-3 (type 1 diabetes mellitus and Hashimoto’s thyroiditis), and noted that patients with PA have an increased risk for various new-onset autoimmune diseases—a risk that persists despite adrenalectomy or mineralocorticoid receptor antagonist treatment—suggesting a possible association between aldosterone excess and autoimmune endocrine gland damage (3).

From an immunological perspective, autoantibodies to the angiotensin II type 1 receptor (AT1R) have been detected in a high percentage of patients with PA, irrespective of underlying etiology. In one study, 75% of patients with idiopathic adrenal hyperplasia and 46% of those with aldosterone-producing adenoma were positive for AT1R-activating autoantibodies (25). These autoantibodies can directly stimulate aldosterone production in adrenal cells and may contribute to the pathophysiology of PA (26). Interestingly, Rossi et al. found that elevated AT1R autoantibody titers did not normalize after adrenalectomy, suggesting that these autoantibodies are not merely a consequence of hyperaldosteronism but may represent a primary immunological abnormality (27). This raises the possibility of shared immunogenetic susceptibility—involving HLA haplotypes (APS-2 is strongly associated with HLA-DR3 and HLA-DR4 (28)) and autoantibody-mediated mechanisms—that may underlie the rare coexistence of PA and APS-2, although a direct causal link cannot be concluded from a single case.

In contrast, the case reported by Puentes et al. described adrenal insufficiency following abdominal aortic aneurysm rupture with prolonged hypotension, attributable to ischemic injury rather than autoimmune adrenalitis (4). Our case is distinct in that adrenal insufficiency was autoimmune, evidenced by hyperpigmentation, Hashimoto’s thyroiditis, and response to glucocorticoid replacement, supporting clinically probable APS-2. The concurrent or sequential manifestation of multiple autoimmune endocrinopathies is well recognized in APS-2, with associated conditions possibly separated by years or occurring simultaneously (29). Our case is unique in that PA and clinically probable APS-2 coexisted, making both diagnosis and treatment challenging.

Therapeutic management required balancing the two conditions’ opposing effects. Spironolactone, a mineralocorticoid receptor antagonist, controlled BP effectively yet worsened hyponatremia. Prednisone-based glucocorticoid replacement was essential for adrenal insufficiency, with close monitoring of electrolytes and BP critical. As reported in PA-APS-3 cases, combined surgical and replacement therapy may work well (3). However, in our patient, medical management with spironolactone and prednisone was the chosen approach due to patient preference and the likely unilateral adrenal hyperplasia subtype of PA.

Limitations: Confirmatory anti-21-hydroxylase autoantibody testing was unavailable due to institutional resource constraints. Cosyntropin-stimulation testing could not be performed because glucocorticoid replacement had already been initiated, which would invalidate assay interpretation. Although clinical findings, low DHEA-S, and therapeutic response strongly support autoimmune Addison’s disease (30), definitive confirmation cannot be achieved. Adrenal vein sampling was declined by the patient, so PA sub-typing remains presumptive.

## Conclusion

In conclusion, this case illustrates primary aldosteronism coexisting with clinically probable autoimmune polyglandular syndrome type 2. In patients with refractory hypertension and unexplained electrolyte disturbances, the possibility of multi-endocrine autoimmunity should be considered, as overlapping endocrine disorders may exert opposing physiological effects that obscure the underlying diagnosis. Moreover, the discordant cortisol–ACTH pattern observed here underscores the inherent lability of ACTH; clinicians must remain vigilant against preanalytical artifacts and interpret endocrine data in conjunction with clinical presentation, cortisol circadian rhythms, and radiographic findings.

## Patient perspective

Following spironolactone-based blood pressure control, prednisone initiation and levothyroxine dose adjustment, the patient reported marked improvement in energy and appetite. She expressed relief at finally having an explanation for her long-standing symptoms, noting that she had previously been told her hypertension and fatigue were age-related. She now understood not only the basis of her chronic fatigue, weight loss, and recurrent hyponatremia, but also the paradoxical phenomenon that spironolactone, though effective for lowering her blood pressure, could aggravate hyponatremia. She acknowledged substantial clinical benefit from the revised therapeutic regimen.

## Data Availability

The original contributions presented in the study are included in the article/supplementary material. Further inquiries can be directed to the corresponding authors.
